# Phylum-wide comparative genomics unravel the diversity of secondary metabolism in Cyanobacteria

**DOI:** 10.1186/1471-2164-15-977

**Published:** 2014-11-18

**Authors:** Alexandra Calteau, David P Fewer, Amel Latifi, Thérèse Coursin, Thierry Laurent, Jouni Jokela, Cheryl A Kerfeld, Kaarina Sivonen, Jörn Piel, Muriel Gugger

**Affiliations:** Commissariat à l’Energie Atomique et aux Energies Alternatives (CEA), Genoscope & CNRS, UMR 8030, Laboratoire d’Analyse Bioinformatique en Génomique et Métabolisme, Evry, France; Department of Food and Environmental Sciences, University of Helsinki, Helsinki, Finland; Centre National de la Recherche Scientifique (CNRS), Aix-Marseille University, Marseille, France; Institut Pasteur, Collection des Cyanobactéries, Paris, France; Department of Plant and Microbial Biology, University of California, Berkeley, CA USA; DOE Plant Research Center, Michigan State University, Michigan, MI USA; Institute of Microbiology, Eidgenoessiche Technische Hochschule (ETH), Zurich, Switzerland

**Keywords:** Cyanobacteria, Secondary metabolite, NRPS, PKS, Diversity, Evolution

## Abstract

**Background:**

Cyanobacteria are an ancient lineage of photosynthetic bacteria from which hundreds of natural products have been described, including many notorious toxins but also potent natural products of interest to the pharmaceutical and biotechnological industries. Many of these compounds are the products of non-ribosomal peptide synthetase (NRPS) or polyketide synthase (PKS) pathways. However, current understanding of the diversification of these pathways is largely based on the chemical structure of the bioactive compounds, while the evolutionary forces driving their remarkable chemical diversity are poorly understood.

**Results:**

We carried out a phylum-wide investigation of genetic diversification of the cyanobacterial NRPS and PKS pathways for the production of bioactive compounds. 452 NRPS and PKS gene clusters were identified from 89 cyanobacterial genomes, revealing a clear burst in late-branching lineages. Our genomic analysis further grouped the clusters into 286 highly diversified cluster families (CF) of pathways. Some CFs appeared vertically inherited, while others presented a more complex evolutionary history. Only a few horizontal gene transfers were evidenced amongst strongly conserved CFs in the phylum, while several others have undergone drastic gene shuffling events, which could result in the observed diversification of the pathways.

**Conclusions:**

Therefore, in addition to toxin production, several NRPS and PKS gene clusters are devoted to important cellular processes of these bacteria such as nitrogen fixation and iron uptake. The majority of the biosynthetic clusters identified here have unknown end products, highlighting the power of genome mining for the discovery of new natural products.

**Electronic supplementary material:**

The online version of this article (doi:10.1186/1471-2164-15-977) contains supplementary material, which is available to authorized users.

## Background

Cyanobacteria are an ancient lineage of morphologically diverse bacteria that fundamentally shaped our planet through the evolution of oxygenic photosynthesis and they continue to play an important role in the global nitrogen and carbon cycles [1, 2]. Cyanobacteria are prolific producers of notorious toxins [3]. These include potent hepatotoxins and neurotoxins such as microcystins, anatoxins and saxitoxins produced by aquatic bloom-forming cyanobacteria around the world. They are also a promising source of natural products with relevance to drug development and biotechnological exploitation [4–6]. The chemical structure of natural products is often characterized from particular cyanobacterial isolates or consortia sampled from the environment and the biosynthetic origins of the major toxins produced by cyanobacteria in marine or fresh waters have now been elucidated [7–10]. However, it is clear that cyanobacteria typically encode additional natural products [11–14].

In a recent effort of better representing the cyanobacterial phylum at the genomic level, we initiated the genetic potential for the secondary metabolite production in Cyanobacteria [1]. This preliminary study confirmed the impressive potential for natural product production across the entire cyanobacterial lineage as 70% of the cyanobacterial genomes contained the polyketide synthase (PKS) and nonribosomal peptide synthetase (NRPS) pathways or hybrids thereof. The NRPS and PKS are two classes of large modular enzymes in which modules incorporate building blocks into the growing chain like in an assembly line. Interestingly, Cyanobacteria dedicated about 5% of their genomes for these pathways, with an average of five NRPS/PKS clusters per genome [1].

The current understanding of the diversification of these pathways is largely based on the knowledge acquired from studies focused on the biosynthesis of few compounds mostly linked to NRPS/PKS pathways. From an evolutionary perspective, some cyanotoxins appeared vertically inherited throughout the phylum *i.e*. microcystin/nodularin family [15], while others such as saxitoxins resulted from multiple horizontal gene transfers (HGT) [16]. On a more global scale, early phylogenetic analyses of these genes acting collectively promoted the importance of HGT to explain their conservation in different bacterial lineages as well as multiple gene duplications and gene loss with vertical inheritance to understand the domain evolution of the PKS pathways [17–20]. Despite the multiplication of chemical characterizations of specific compounds coupled with genomic investigation and examples from various bacterial lineages, the biosynthetic origins of the natural products mostly remain unknown. On the other hand, there is a true need to understand the way these pathways evolve notably to produce natural product-like by joining functionally subclusters and enzymes through construction of novel artificial biological pathways.

Here we performed a large-scale analysis of cyanobacterial natural product pathways by combining genomic data with genetic information for biosynthesis of specific compounds as well as on genetic conservation of the domains of these genes. Several additional gene cluster families were distinguished, among which some of the compounds produced are likely specialized for basic cell functions, like chelating iron from surrounding environment or contributing to the final maturation of the heterocyst. This first phylum-level investigation allowed identification of different evolutionary forces that shape the metabolic diversity of natural products in an ancient lineage of bacteria. In addition to these insights, our data provide a genetic framework for the chemical characterization of these compounds for biotechnological and pharmaceutical applications.

## Results

### Identification of NRPS/PKS pathways

Genomic analysis identified 452 biosynthetic gene clusters including 190 NRPS, 162 PKS, and 100 hybrid gene clusters encoding both NRPS and PKS enzymes from 89 cyanobacteria out of the 126 genomes of the CyanoGEBA dataset covering the diversity of the phylum [1] (Table 1). The PKS contain at least one ketosynthase domain and belong to type I modular systems, subdivided into cis- and transacyl transferase, and type I iterative PKSs, as well as type III PKSs [21]. Furthermore, various mixed polyketide pathways were found such as type I/type III PKSs. The NRPS contain either a typical NRPS with, minimally, adjacent condensation and adenylation domains or a NRPS-like lacking of condensation domain [22, 23]. Hybrids contain PKS linked to NRPS modules, which results in the production of polyketide–peptide hybrid metabolites.

**Table 1:** **NRPS/PKS gene clusters and cluster families**

Types of clusters	No. of clusters	No. of clusters of		Orphan	No. of CF
		Known product (CF)	Unknown product (CF)		
PKS	162	61 (6)	48 (13)	53	72
Hybrid	100	13 (7)	36 (14)	51	72
NRPS	190	17 (6)	52 (15)	121	142
Total	452	91 (19)	136 (42)	225	286

Gene clusters encoding NRPS, PKS and hybrid NRPS/PKS were found in 89 out of 126 cyanobacterial genomes (Additional file 1: Table S1). Several gene clusters were shared among Cyanobacteria, forming cluster families (CFs). Some CFs correspond to biosynthetic pathways of known products, whereas others without associated products were recovered using BLASTP and a transitive link criterion to build families. The other half of the gene clusters were orphans, each forming a putative family by its own.

To further analyse this exceptionally rich metabolic gene set, we aimed to identify groups of gene clusters related to each other. We combined similarity results of the comparisons of protein sequences of the 452 clusters against each other with synteny conservation parameters in order to gather similar pathways into gene cluster families (CF) that potentially encode for megasynthetases involved in the biosynthesis of closely related metabolites. This method allowed the identification of CF of known natural product pathways, such as anabaenopeptin and nodulapeptin clusters involved in the synthesis of large family of cyclic hexapeptide with a conserved D-Lys and ureido linkage [24]. We also obtained one CF gathering microcystin and nodularin pathways, both involved in the biosynthesis of peptides harbouring the characteristic C20 amino acid, 3-amino-9-methoxy-2,6,8-trimethyl-10 phenyl-4,6-decadienoic acid (Adda) [25]. In addition, clusters of different lengths but involved in the biosynthesis of variants of a compound, *i.e*. clusters of aeruginosin of 13 to 25 kb-long with an amino-acid sequence identity as low as 55%, were grouped within the same CF. This method also permitted the identification of families of related gene clusters when the latter were fragmented on different contigs as one might expect in unfinished genomes.

### Pathway diversity in Cyanobacteria

**Figure 1:**
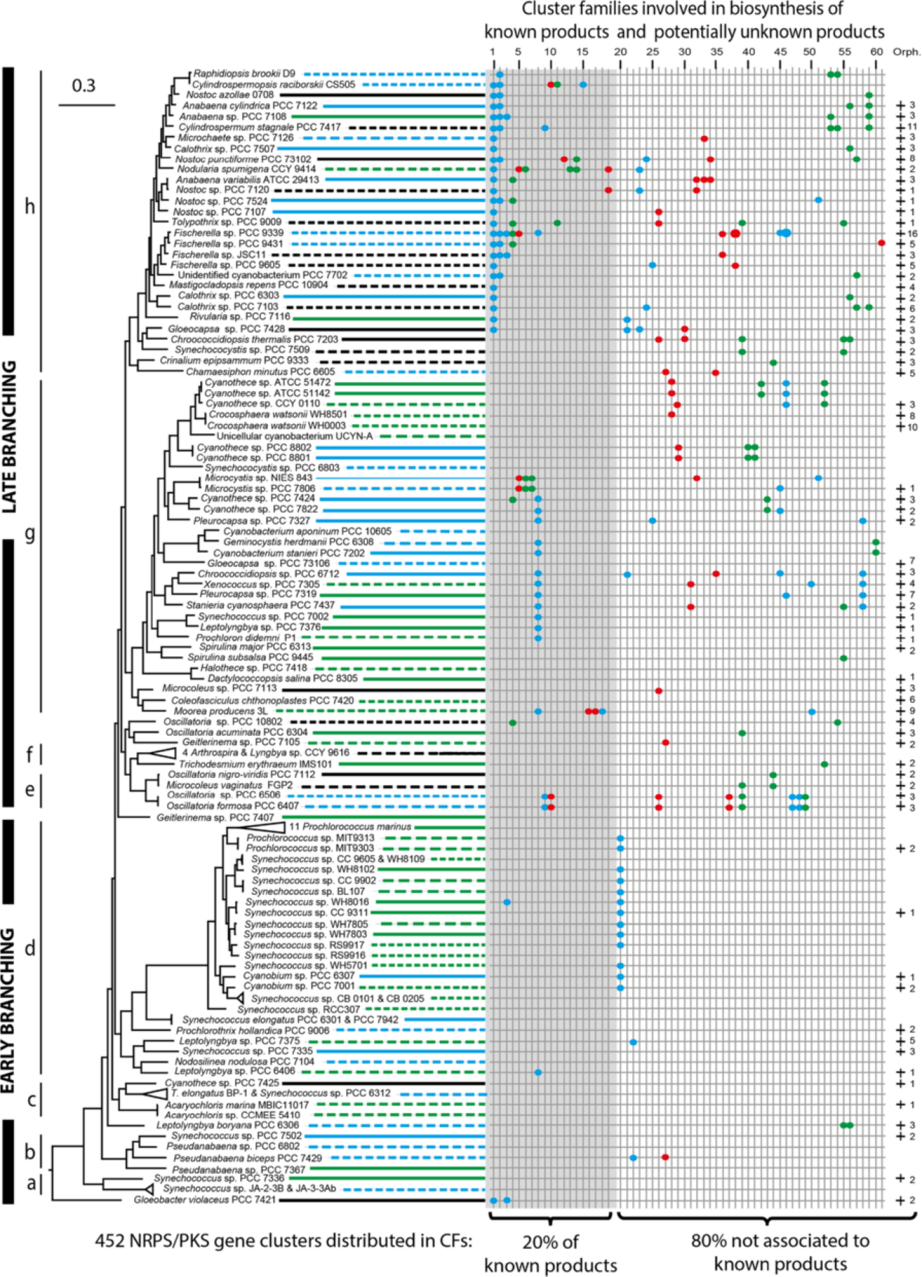
**Distribution of shared and orphan NRPS/PKS gene clusters detected in Cyanobacteria.** The species tree was generated by a concatenation of twenty-nine conserved proteins using a Maximum Likelihood method. The clades a to h of the phylogenetic tree are supported by a bootstrap of ≥70%. The species tree is connected to the distribution pattern by lines. The lines are plain for complete genome and dashed for unfinished genomes and indicate the habitat of the strains: blue for fresh water, green for marine and black for other. On the distribution pattern, the cluster families involved in the biosynthesis of known product are first indicated in the grey shadowed area from CF-1 to CF-19, followed by the shared ones and encoding potentially unknown product from CF-20 to CF-61 with a white background while the last column indicates the number of orphans. The PKS clusters are indicated in blue, hybrid in red and NRPS in green. Each cluster is indicated by a dot, except for two gene clusters present in double copy in the genome of PCC 9339 that are indicated by a larger dot. The colored clusters on the same vertical line are related to each other and define a family of clusters. Details on the species tree and on the clusters and cluster families are available on Additional file 2: Figure S1 and Additional file 1: Table S1, S2 and S3.

**Figure 2:**
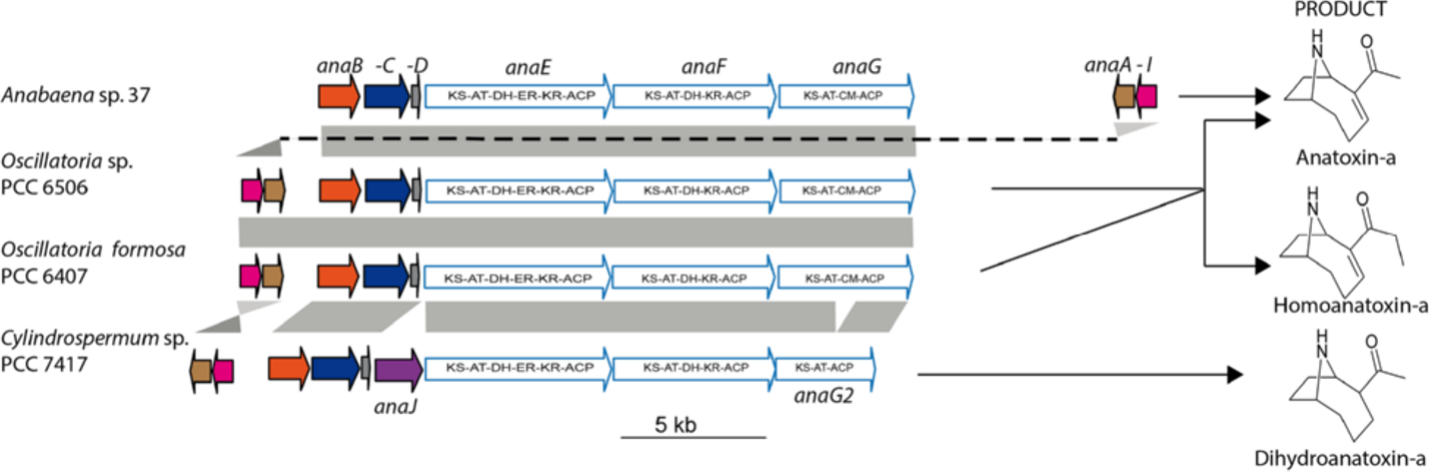
**Anatoxin-a biosynthetic gene cluster (CF-9) and produced variants of in PCC 7417.** Anatoxin-a pathway identified in the genome of *Cylindrospermum* sp. PCC 7417 compared to homologous gene clusters from anatoxin-a producing cyanobacteria [8, 26, 27]. Genes with corresponding functions and domain organization are colored the same and connected by grey areas: *anaA*, proline adenylation, *anaB*, proline deshydrogenase, *anaC*, Type II thioesterase; *anaD*, acyl carrier protein, *anaE*, *anaF* and *anaG* are modular type I polyketide synthase with KS, β-ketoacyl synthase; AT, acyltransferase; KR, ketoacyl reductase; ACP, acyl carrier protein; DH, dehydratase; ER, enoyl reductase and CM, *C*-methyltransferase. The cyclase, named here *anaI*, is systematically associated to the anatoxin-a pathway. In addition, the last PKS *anaG2* in PCC 7417 lacks the methyltransferase, and an oxidoreductase *anaJ* was detected. The transposase is present in the surrounding of the cluster only in PCC 6506. The detection of dihydroanatoxin-a from PCC 7417 is presented in Additional file 2: Figure S2.

We mapped the distribution of the CFs onto the species tree of Cyanobacteria to track for their diversity and their distribution in the phylum (Figure 1, Additional file 1: Table S1). Interestingly, only 20% of the gene clusters could be assigned to the described biosynthetic pathway of a natural product belonging to a group of known chemical compounds (Additional file 1: Table S2), indicating a rich diversity of new chemical scaffolds. These assigned groups comprise 91 gene clusters belonging to 19 CFs, which encode multi-enzymatic proteins involved in the synthesis of well-described bioactive compounds, such as protease inhibitors, UV sunscreen agents, and toxins (Table 1, Additional file 1: Table S3). Most of these gene clusters exhibit a patchy taxonomic distribution throughout the cyanobacterial lineage. But there were two noticeable exceptions of PKS pathways found in genomes of clades g and h, respectively, containing all types of cyanobacterial morphologies: (i) the CF-8 predicted to be involved in the production of hydrocarbons; (ii) the CF-1 involved in the biosynthesis of polyunsaturated fatty acids (PUFA). Also, the genomic analysis combined with metabolic characterisation highlighted more variability in the biosynthetic clusters of well-known toxins as examplified by anatoxin-a (CF-9) and microcystin (CF-5). The CF-9 gathers three clusters coding for anatoxin-a biosynthesis identified in four strains. The one present in the potent neurotoxin producing strain *Cylindrospermum* sp. PCC 7417 contained in addition an oxidoreductase (*anaJ*) and a truncated *anaG* leading to the production of dihydroanatoxin-a as the major variant (Figure 2, Additional file 2: Figure S2). The microcystin biosynthetic cluster in *Fischerella* sp. PCC 9339 and the detection of microcystin-LR in this strain confirm the presence of this hepatotoxin family in the most complex morphotypes of the Cyanobacteria (Additional file 2: Figure S2).

Regarding the 80% of the 452 gene clusters associated with the biosynthetic pathway of unknown compound, 136 of the gene clusters were grouped into 42 additional CFs based on their similarity and shared gene content by applying the same classification (Table 1, Additional file 1: Table S3). The remaining 225 gene clusters not known to be involved in particular compound biosynthesis and not related to each other were considered to be unique orphan gene clusters, each of them representing putatively independent CFs (Table 1). Among the uncharacterized CFs, the PKS CF-20 was found widely distributed in the marine or fresh water picocyanobacteria of the clade d, which appeared rather depleted of other NRPS/PKS gene clusters.

Thus, the initial 452 gene clusters grouped into 286 CFs. As for several examples of NRPS/PKS clusters, these CFs are potentially involved in biosynthesis pathways. However, some pathways may not be functional while others produce notorious compounds or even known compounds for which the biosynthesis has not yet been investigated. A rank-abundance curve describing the distribution of the CF among our dataset reveals that most of the CFs sharing highly similar gene content in synteny are spread only into 2 to 3 genomes, and one up to 25 strains (Additional file 2: Figure S3). The long tail consisting of orphan CFs unique to each genome evidenced a high diversity of the NRPS/PKS gene clusters in Cyanobacteria and a largest one to discover with function of further sequencing effort. The 225 orphan CFs are distinct from the shared CFs by their size as they are limited to their single version of NRPS/PKS genes without any tailoring enzyme. But despite their smaller mean size, their number is likely not overestimated as they are similarly occurring in complete and unfinished genomes (Additional file 2: Figure S3).

### Pathway distribution in the cyanobacterial phylum

**Figure 3:**
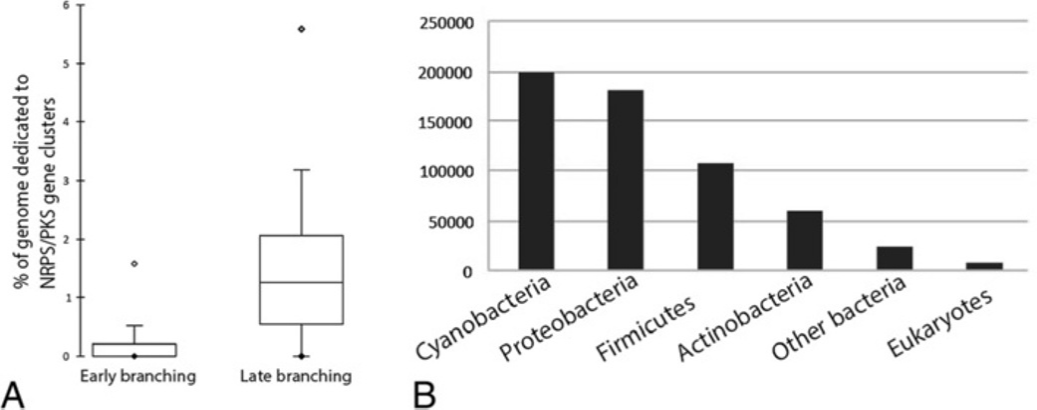
**Proportion of NRPS/PKS genes clusters in cyanobacterial genomes and their closest homologs. A**. Percent of genome dedicated to encode NRPS/PKS gene clusters highlights the burst in the late-branching clades in comparison to the early-branching ones of the phylogenetic species tree (Figure 1), **B**. Distribution of the BLAST hits of the 2668 proteins contained in the 452 clusters compared to the non-redundant database of NCBI (identity > =30% and e-values <10e^-20^).

The analysis of the gene cluster distribution at the cyanobacterial phylum level showed that there are more genomes harboring NRPS/PKS gene clusters in the late branches of the species tree (65 out of 75 genomes) than in the early ones (24 out of 51 genomes). In addition, the percentage of genome devoted to encode these gene clusters is higher in the late-branching part of the cyanobacterial lineage (Figure 3A). On average, there are 6.2 clusters per genome in the late branches compared to 2 clusters per genome in the early ones (403 clusters in late-branching cyanobacteria *versus* 49 clusters in early-branching ones).

Up to 30 of the 61 NRPS/PKS gene cluster families are shared among closely related strains as exemplified by the CF-28, CF-42, CF-46 and CF-52 present in *Cyanothece* sp. ATCC 51142 and ATCC51472, and the CF-9, CF-10, CF-26, CF-37, CF-39 and CFs-47-49 found in *Oscillatoria* spp. PCC 6407 and PCC 6506 (Figure 1), and indicated a vertical inheritance of the gene clusters from their common ancestors. The simultaneous occurrence of several common CFs was found between groups of two to three cyanobacteria only. At the phylum level, only three PKS CFs (CF-1, CF-8 and CF-20) are more widely shared by cyanobacteria of the same phylogenetic clades, e.g. d, g, and h, coherent with a vertical inheritance and some subsequent losses. On the other hand, all ten CFs of *Crocosphaera watsonni* WH0003, the six CFs of *Coleofasciculus chthonoplastes* PCC 7420 or the only CF of *Acaryochloris marina* MBIC11017 located on one of its plasmids were present only in those genomes of the analysed dataset. Therefore, we performed a Hierarchical Clustering analysis of the 286 pathways (Additional file 3), which indicated a species clustering not coherent with the species phylogeny (Figure 1), and thus confirms that the NRPS/PKS gene clusters widely spread in this dataset are likely not vertically inherited as a whole in the phylum.

BLAST analyses of the proteins of the 452 clusters against the non-redundant database of NCBI indicated that the closest homologs are always found within the Cyanobacteria. However, 89% of the proteins of the clusters also have homologs in other bacteria well-known for their NRPS and PKS content such as the Proteobacteria, Firmicutes or Actinobacteria [28, 29] (Figure 3B). While the NRPS and PKS gene clusters are widespread in several bacterial phyla, 11% of the biosynthetic proteins encoded in the 452 clusters have hits only in the cyanobacterial phylum. These cyanobacteria-specific proteins correspond to non-NRPS/PKS proteins composing the clusters, suggesting that the specificity of the cyanobacterial NRPS/PKS clusters relies on accessory proteins such as tailoring enzymes involved in the maturation of the produced peptide.

### Evolution of the pathways

**Figure 4:**
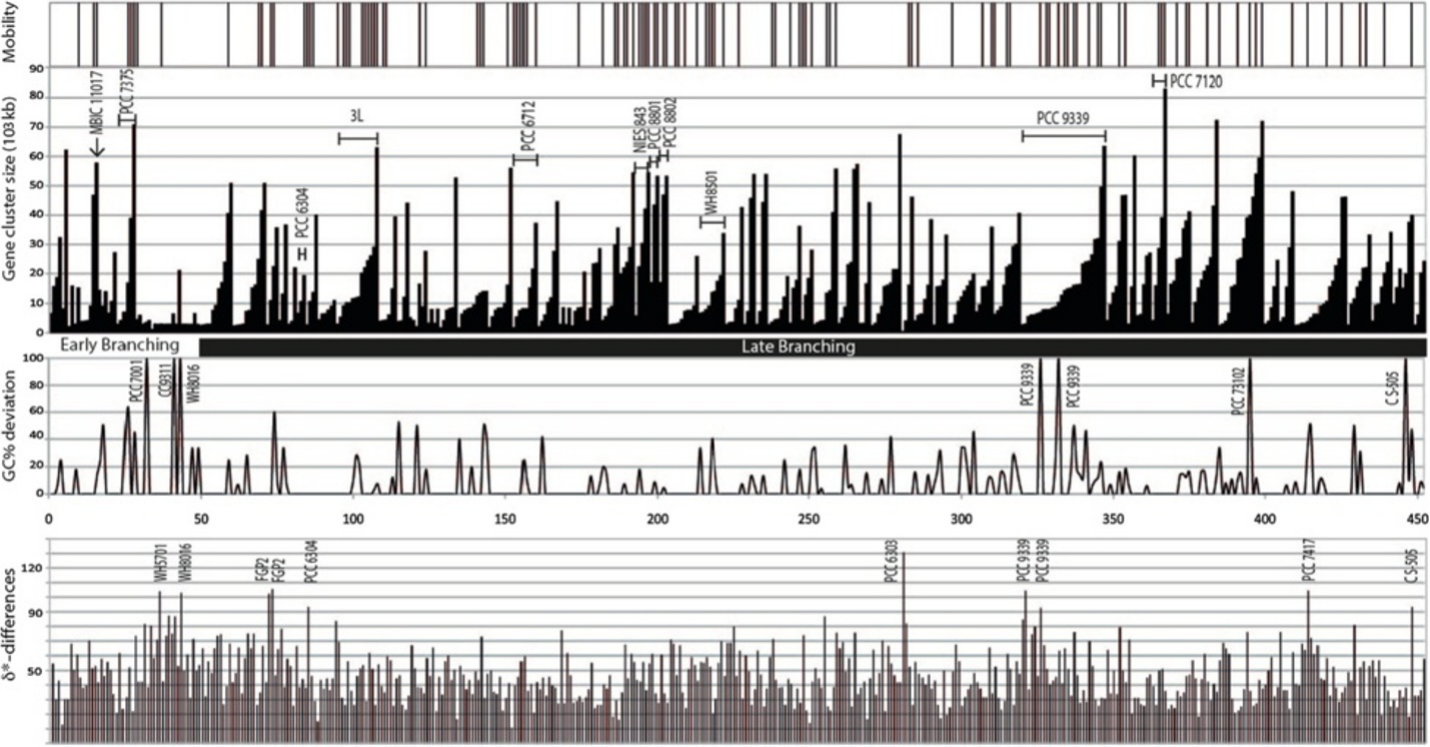
**NRPS/PKS genes clusters in Cyanobacteria: mobility, gene cluster size, proportion of GC% deviated genes and dinucleotide average absolute relative abundance difference.** The size of the 452 gene clusters is indicated for each strain with their corresponding percentage of genes presenting below a GC% deviation and dinucleotide average absolute relative abundance difference (δ*-differences) and above associated with presence of mobility traces in their genomic context. The genomes are ordered accordingly to the phylogenetic tree presented in Figure 1.

The impact of HGT on the evolution of these pathways at the phylum level is difficult to estimate due to the complexity of our dataset. Dinucleotide signature analysis of the clusters distinguished 132 clusters with atypical genomic signature (δ*-differences ≥55 [30], indicated in the lowest pattern of Figure 4, Additional file 1: Table S1). Among them, 10 are particularly biased (δ*-differences ≥90 [30]), suggesting an external acquisition from a distantly related organism, notably CF-3 in *Synechococcus* sp. WH8016, CF-8 in *Fischerella* sp. PCC 9339, CF-10 in *Cylindrospermopsis raciborskii* CS-505, CF-20 in WH5701 and 6 orphan clusters in diverse cyanobacterial morphotypes. In addition, CF-3 in *Synechococcus* sp. WH8016 and CF-8 in *Fischerella* sp. PCC 9339 showed a GC% deviation on their full length (Additional file 1: Table S1).

Moreover, 4% of the gene clusters, with sizes up to 58 kb, are present on plasmids (Additional file 1: Table S1) representing potential vectors for HGT. Also a detailed examination of the genomic context of the NRPS/PKS gene clusters indicated that 26% are surrounded by or contain genes encoding mobile elements (*i.e*. transposases, phages, integrases) potentially involved in HGT, which impact 47 of the 89 genomes harbouring these gene clusters. It concerned 63% of the genomes in the late-branching clades of the phylogenetic cyanobacterial tree and only 25% in the early-branching clades (Figure 4). It has to be noted that most of the mobile elements identified are highly degraded and correspond likely to gene remnants. Most, if not all, of the gene clusters of eleven genomes (*Acaryochloris marina* MBIC 11017, *Leptolyngbya* sp. PCC 7375, *Oscillatoria acuminata* PCC 6304, *Moorea producens* 3 L, *Chroococidiopsis* sp. PCC 6712, *Microcystis* sp. NIES-843, *Crocosphaera watsonii* WH8501, *Cyanothece* sp. PCC 8801 and PCC 8802, *Fischerella* sp. PCC 9339, *Nostoc* sp. PCC 7120, indicated in Figure 4) are surrounded by traces of mobile elements in their genomic context or are present on plasmid. Altogether, 126 clusters have mobility traces and/or are located on plasmids, among which 37 clusters showed also an atypical dinucleotide signature. Interestingly, 36 of those are occurring in the genomes of the late-branching lineages.

**Figure 5:**
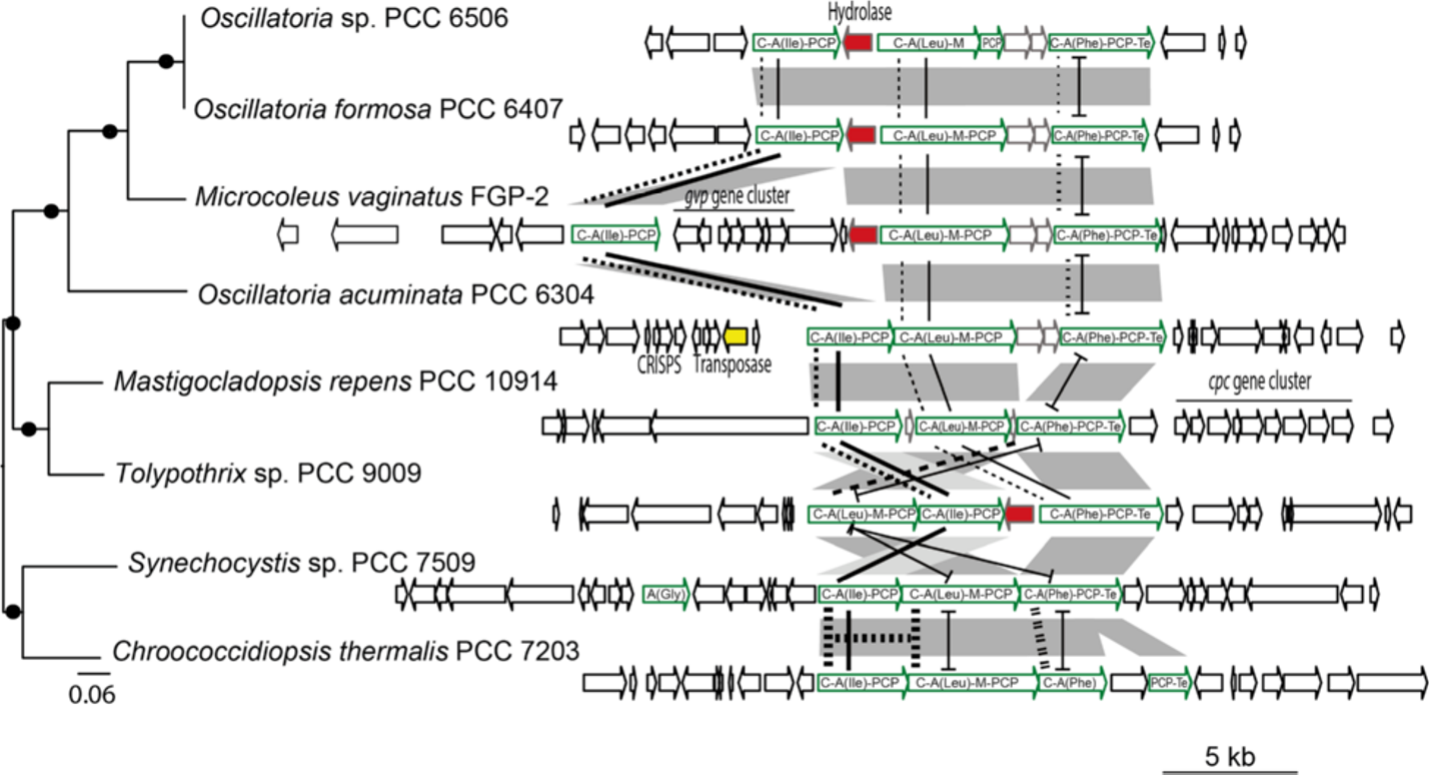
**Evolution of the CF-39 gene cluster.** The CF-39 gene clusters are ordered according to the species phylogeny. The gene clusters and their flanking sequences are pictured: NRPS genes are surrounded in green with their domain composition detailed as well as the substrate specificity of A domain (parentheses), the conserved hydrolase gene is indicated in red, transposase one in yellow, and other genes are pictured in white and annotated when their function is known. C and A domain phylogenies were reported as lines that link the homologous domains by the same type of lines.

However, HGT might not be the main driving force acting on the evolution of NRPS/PKS gene clusters in Cyanobacteria. An analysis of the phylogenies of the NRPS condensation (C) and PKS ketoacyl synthase (KS) domains supported complex evolution with a domain diversification that suggests the incorporation of diverse substrates (Additional files 4, 5). While the phylogeny of C domains found in our dataset (Additional file 2: Figure S4) showed clustering into previously described C-domain subtypes, the phylogenetic analysis of KS domains revealed the supported clustering of all KS involved in PUFA and enediyne biosynthesis (Additional file 5; see below). A close examination of the clusters composing our CFs highlights different evolutionary scenarios. The clusters of CF-9 (anatoxin-a), CF-20 or CF-32 showed a conservation of gene order and content (Figure 2, Additional file 2: Figure S5). On the contrary in CF-5, CF-26, and CF-39 (Figure 5, Additional file 2: Figure S2, S5), the clusters went through more complex evolution schemes involving gene duplication, indels and/or inversions as well as domain deletion/substitution. The KS and C domains phylogenies of CF-39 showed strong synteny conservation in filamentous cyanobacteria (*Oscillatoria* spp. PCC 6407 and PCC 6506 and *Microcoleus vaginatus* FPG-2 in Figure 5), counter-balanced by events of gene shuffling, domain recombination and duplication during the evolution in heterocystous and unicellular cyanobacteria (*Tolypothrix* sp. PCC 9009, *Synechocystis* sp. PCC 7509 and *Chroococidiopsis thermalis* PCC 7203 in Figure 5).

### Further roles for secondary metabolites in Cyanobacteria

Most secondary metabolite pathways are not linked to the cyanobacterial lifestyle in their environments e.g. marine *vs* fresh water, but some obviously benefit to the harbouring organisms to cope with their habitats. In our dataset, two examples covering several cyanobacterial genomes illustrate such benefice.

**Figure 6:**
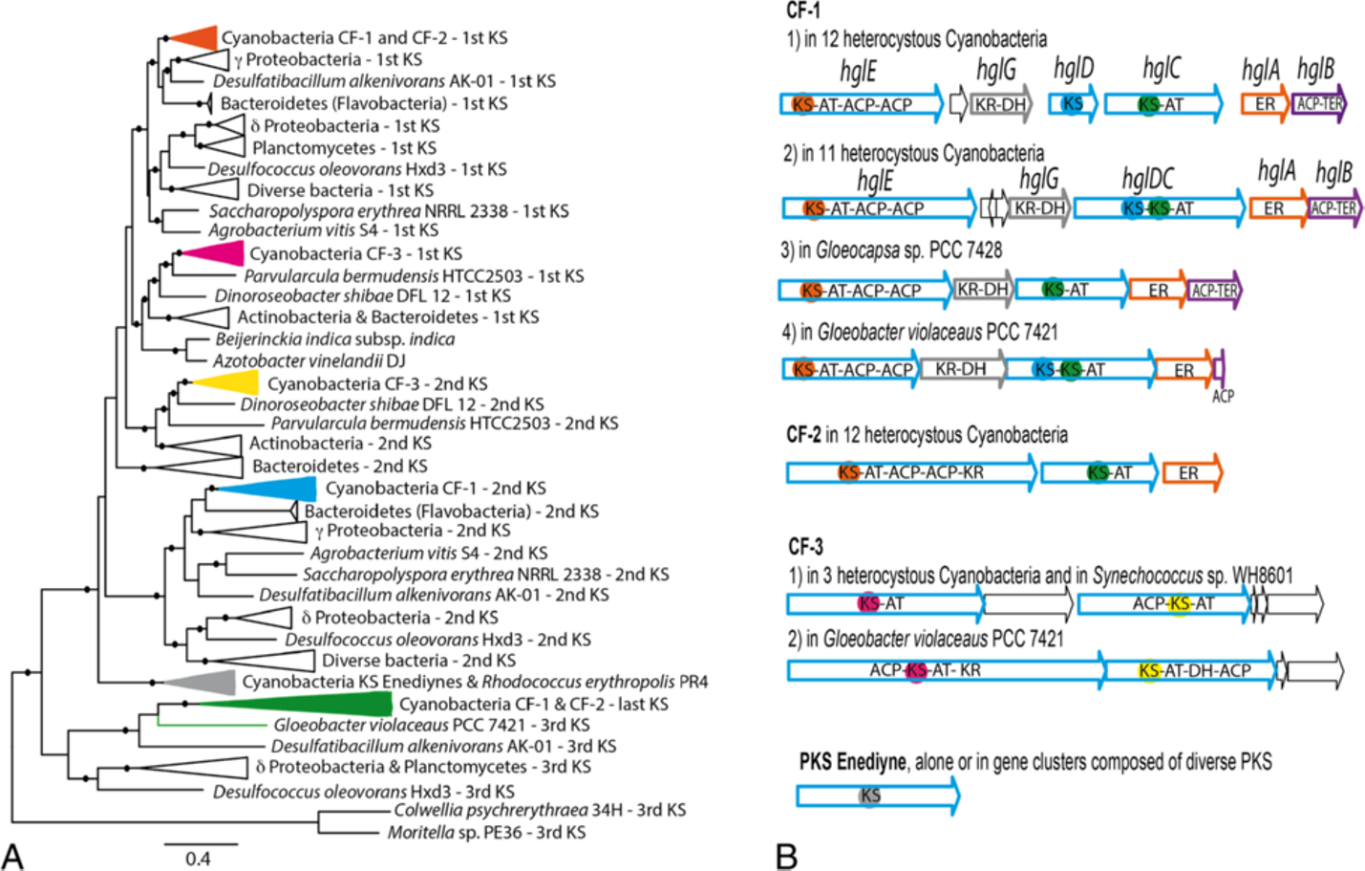
**Phylogeny of bacterial ketoacyl synthase domains and their distribution in cyanobacterial PKS PUFA gene clusters. A**. Maximum likelihood phylogenetic tree of bacterial KS based on 235 conserved amino acids from 217 gene clusters encoding secondary lipids biosynthesis and enediynes. The clades of the phylogenetic tree supported by a bootstrap of ≥70% are indicated by a black dot. **B**. PKS gene clusters dedicated to PUFA and enediynes in Cyanobacteria. The background color of each KS refers to the corresponding clade of the adjacent phylogenetic tree. PKS genes are indicated in blue, whereas homologous genes are indicated by similar colors. KS, ketoacyl synthase; AT, acyltransferase; KR, ketoacyl reductase; ACP, acyl carrier protein; DH, dehydratase; ER, enoyl reductase; TER, thioester reductase.

The first example concerns the PKS PUFA gathered in CF-1 which are involved in the production of heterocyst glycolipids as the last maturation step of the heterocyst rendering it impermeable to oxygen produced by adjacent cells, and thus, able to fix atmospheric nitrogen [31, 32]. The evolutionary data of the PKS PUFA within Cyanobacteria clearly supported their vertical inheritance, as the KS PUFA were forming a supported and large monophyletic clade with the enedyine KS in the KS phylogenetic tree (Additional file 5). Comparison of these KS with homologs from other bacteria [33] supported six cyanobacterial KS clades (Figure 6A). The first and second KS of the CF-1, CF-2 and CF-3, highlighted in orange, pink, yellow and blue of the phylogenetic tree and in the gene cluster schemes (Figure 6), emerged by duplication. Moreover, they are closely related to their counterparts in other phyla, so that the first KS of CF-1 and CF-2 (orange clade) and the second KS of CF-1 (blue clade) are related to those in γ-Proteobacteria and Flavobacterium, while the two first KSs of CF-3 (pink and yellow clades) are more closely related to those in Actinobacteria. The KS enediyne clade in grey is basal to the monophyletic group comprising the first and second KS of CF-1, CF-2 and CF-3. Finally, the last KS of the CF-1 and CF-2 represented in green colour diverged earlier from these, as did other terminal KS from PUFA clusters in Planctomycetes and δ-Proteobacteria.

**Figure 7:**
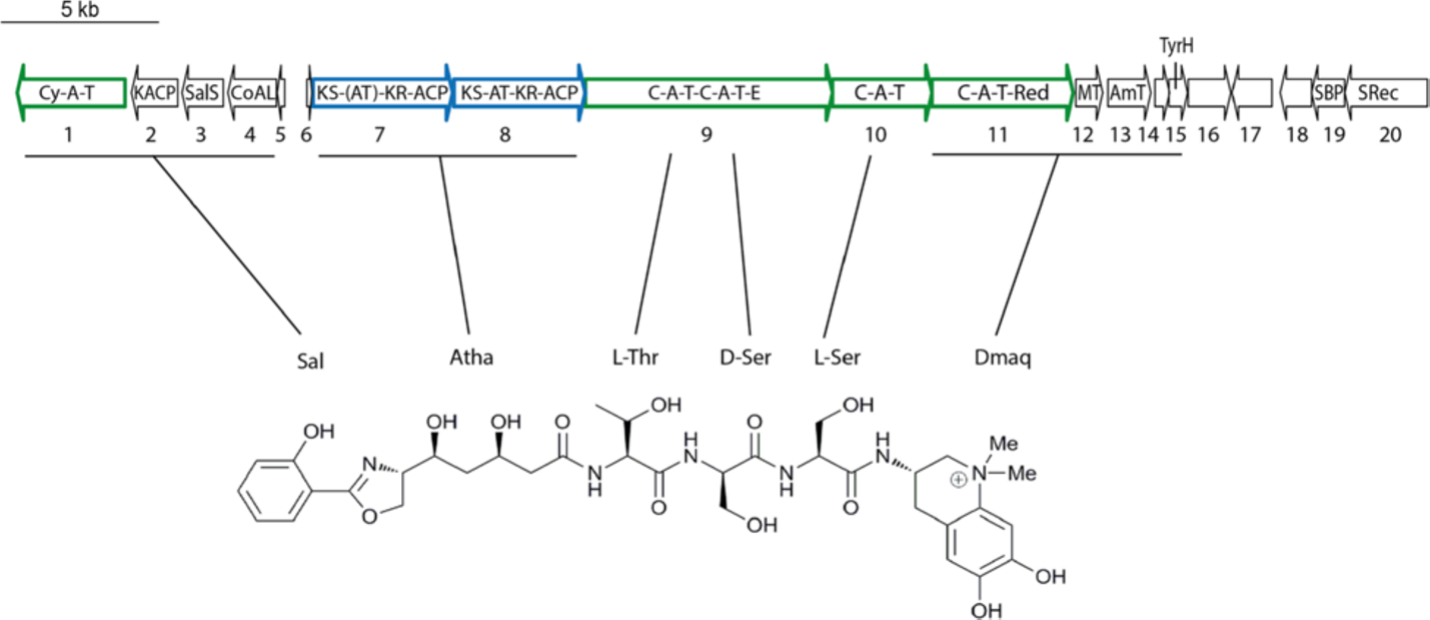
**Model for the formation of the siderophore anachelin 1.** Hybrid gene cluster of *Anabaena cylindrica* PCC 7122 with a NRPS architecture in agreement with the assembly of the characteristic three hydrophilic amino acids (L-Thr-D-Ser-L-Ser) and the two units responsible for binding iron (Atha and Dmaq) of the anachelin 1 structure [37]. In addition, PKS tailoring and transport-related genes are present. Symbol: Cy, cyclisation; A, adenylation; T, thiolation; KACP, KSIII acyl carrier protein; SalS, salicylate synthase; CoAL, salicylyl CoA/PCP ligase; KS, β-ketoacyl synthase; AT, acyltransferase; KR, ketoacyl reductase; ACP, acyl carrier protein; E, epimerase; Red, reductase; MT, methyl transferase; AmT, aminotransferase; TyrH, putative tyrosine hydrolase; SBP, siderophore binding protein; Srec, siderophore receptor; Sal, salicylic acid; Atha, 6-amino-3, 5, 7-trihydroxyheptanoic acid; Thr, threonine; Ser, serine; Dmaq, 1,1-dimethyl-3-amino-1,2,3,4-tetrahydro-6,7-dihydroxyquinolinium. The NRPS genes are indicated in green, the PKS genes are in blue.

The second example obtained from the examination of the genomic context of the 452 gene clusters resulted in the identification of 62 gene clusters containing genes related to siderophore transport systems and suggesting the involvement of NRPS/PKS gene clusters in siderophores production (Additional file 1: Table S1). This findings was unexpected, as only a few siderophores were previously characterized in Cyanobacteria, and mostly linked to the biosynthesis of ribosomally synthesized and post-translationally modified peptides (RiPPs) like for synechobactin A-C in *Synechococcus* sp. PCC 7002 and schizokinen in *Anabaena* species [34]. We analysed a 34 kb-long NRPS/PKS gene cluster present in *Anabaena cylindrica* PCC 7122 potentially involved in biosynthesis of anachelin 1 (Figure 7), supporting the wider siderophore potential hypothesis in Cyanobacteria. The catecholate siderophore anachelin is a peptide alkaloid initially characterized from a non-axenic co-identical strain of PCC 7122, *Anabaena cylindrica* CCAP 1403/2A, and confirmed in *Anabaena cylindrica* NIES 19 [35, 36]. This NRPS/PKS gene cluster in PCC 7122 comprised 20 genes with a NRPS architecture in agreement with the assembly of the characteristic three hydrophilic amino acids (L-Thr-D-Ser-L-Ser) and the two units responsible for binding iron (Atha and Dmaq) of the anachelin 1 structure as sketched from earliest structural study [37]. In addition, the genetic analysis allowed identifying the presence of genes 1 to 4 as candidates for the biosynthesis of the salicylate starter unit, genes 7 and 8 participate in the Atha biosynthesis (note the AT domain of the first PKS should be inactive due to an absent active site-motif), genes 9 and 10 encode L-Thr-D-Ser-L-Ser, genes 11-15 encode the Dmaq production, followed by genes 16 to 20 which encode an efflux protein, a thioredoxin-like protein, a siderophore-binding protein and a siderophore receptor respectively. In addition to the anachelin cluster in *Anabaena cylindrica* PCC 7122, we identified in the *Nodularia* sp. CCY 9414 genome an ortholog to clusters encoding compounds with siderophore activity in *Nostoc* sp. PCC 7120 [38] and in *Agrobacterium tumefaciens* C58 [39]. This *in-silico* analysis allows predicting a widespread potential of siderophores not previously anticipated, and implies iron uptake mechanisms to be further explored for Cyanobacteria.

## Discussion

Cyanobacteria are a prolific source of natural products, many of which have complex chemical structures [4, 6, 40]. The large-scale genomic analysis at the phylum-level presented here shows an impressive genetic diversity underlying known and cryptic cyanobacterial metabolism. Indeed, the 286 distinct NRPS/PKS gene cluster families found in this dataset will increase as other already known pathways described in previous works on Cyanobacteria are not represented in the present study. Often highlighted through human health hazard point of view, the clusters encoding major toxins, protease inhibitors and other known bioactive compounds are not predominant in these pathways. Most of the CFs seems to be spread in various groups throughout the phylum, but closely related cyanobacteria shared also similar CF patterns [41, 42]. In our dataset, only three PKS pathways were more largely disseminated within a given group of cyanobacteria (CF-20 in picocyanobacteria of clade d, CF-8 in unicellular, baeocystous unicellular and one filamentous of the clade g, and CF-1 in heterocystous of clade h) coherent with a vertical inheritance and some subsequent losses, and presumably giving them a certain benefit. However, the diversification and the large distribution of the NRPS/PKS gene clusters are inconsistent with global vertical inheritance followed by repeated losses in the current lineages as observed for some of the toxins [15].

Different mechanisms impacted the evolution of these gene clusters in Cyanobacteria shown by the CF-1 and CF-8 largely distributed in a given clade but also spread discretely in other clades. CF-1 was vertically inherited from the root to the clade h to enable spatial nitrogen fixation to heterocystous cyanobacteria. On the contrary, the CF-8 largely spread in clade g has been inherited partially in *Fischerella* sp. PCC 9339 by HGT. Indeed, the lack of the two last enzymes of the CF-8 pathway in this heterocystous strain suggests the non-production of hydrocarbon. Thus, in addition to CFs vertically inherited, others presented a more complex evolutionary history.

We noticed only a few obvious HGT in Cyanobacteria and mainly the lack of gene context conservation in any of the presently defined CFs contrarily to the observed exchange of pathways incorporated into genomic islands for *Salinospora* [43]. Thus, despite genomic islands in one picocyanobacterial group [44] and 26% of the genomic context of our pathways with degraded mobile elements, there is no insertion hotspot facilitating the incorporation of NRPS and PKS genes in Cyanobacteria.

We observed a clear burst of NRPS/PKS pathways in the late-branching Cyanobacteria, which devote a larger part of their genome to these pathways. In addition, about 9% of pathways in the late-branching cyanobacteria have mobility traces and/or are located on plasmids and show a deviation in their dinucleotide signature. Mobile elements might have allowed HGT and/or recombination events. This suggests also that HGT events explain partly the burst of NRPS/PKS gene clusters in the late-branching cyanobacterial clades, which is in agreement with the increase of gene number in the genomes of this lineage [45]. However, the highly degraded state of the mobile elements in the CF’s surroundings suggests also that many of these events were ancient.

Distantly related cyanobacteria present strongly conserved CFs such as CF-9 for anatoxin biosynthesis found in *Oscillatoria* spp. PCC 6407 and PCC 6506 and in *Cylindropsermum stagnale* PCC 7417, or CF-32 present in *Nostoc* sp. PCC 7120, *Anabaena variabilis* ATCC 29413 and *Microcystis* sp. NIES-843, but contain also rearranged CFs that underwent drastic gene shuffling events such as CF-5 for microcystin synthesis in *Microcystis* sp. PCC 7806 and *Fischerella* sp. PCC 9339 and CF-39 in unicellular, filamentous and heterocystous cyanobacteria. Moreover, the tailoring enzymes involved in the maturation of the peptide seem to be more specific to Cyanobacteria than the NRPS/PKS genes. Therefore, gene shuffling combined to specific tailoring enzymes participated to the chemical originality of secondary metabolites in Cyanobacteria and could explain the diversification of these pathways in the whole phylum.

The adaptation and fitness of bacteria depends on their genomic potential for providing various strategies to cope with stressful and changing environments. The ecological function of secondary metabolites is unclear despite continual expression of some clusters, which appear more involved in the physiology of the producing organism [46]. In this dataset also, we evidenced NRPS/PKS gene clusters that might have alternative and adaptive functions notably in heterocystous cyanobacteria to boost their primary metabolism with specific PKS PUFA pathways dedicated to heterocyst glycolipids. Indeed, as the CF-1 and CF-3 clusters are present in *Gloeobacter violaceaus* PCC 7421 at the root of the cyanobacterial phylum, and the KS of these two CFs emerged the same way than in other bacteria, it is likely that these clusters predate the Cyanobacteria. CF-1 was subsequently kept in the heterocystous clade for the heterocyst maturation and the advantage of nitrogen fixation without inhibition of oxygen resulting from photosynthesis. Further, the heterocystous cyanobacteria developed the CF-2 by module loss from CF-1 while the CF-3 was also independently acquired in the marine *Synechococcus* sp. WH8601 from heterocystous cyanobacteria, as supported by domain phylogeny, deviated dinucleotide signature and GC%.

The abundance of pathways putatively encoding siderophores identified in this study also favours this ecological function hypothesis. Considering the low solubility of iron in aerobic environments and the iron rich photosynthetic apparatus of cyanobacteria, diverse strategies for iron chelation could play a role in bloom formation in iron-limited marine and fresh waterbodies [47].

Even though modular NRPS/PKS megasynthases appear like an energetically expensive solution for producing small secondary metabolites, they allow the incorporation of non-protein substrates as well as facile evolution of product diversity [20]. Expansion of NRPS/PKS clusters during evolution of Cyanobacteria resulted in diversification at the genetic level, as basis for the observed chemical diversity of the bioactive metabolites. Previous studies on individual cyanobacterial groups have demonstrated that genomic information can enable the discovery of unanticipated biosynthetic enzymology and compound types [48–52]. Therefore, the identification of shared pathways at the phylum level presented here will serve as a valuable foundation for further metabolic discoveries, particularly in the frame of mass spectral networking analysis [53–55].

## Conclusions

Genomic analysis of secondary metabolism of Cyanobacteria, which is the first conducted at phylum-wide level, reveals the potential of these microbes to recombine, diversify and spread modular NRPS/PKS gene clusters encoding a multitude of compounds. The 286 cluster families identified in this study are representing certain of the biosynthetic pathways of known cyanobacterial compounds, highlighted several new conserved pathways within Cyanobacteria, and reveal a large number of orphan pathways with high drug discovery potential. The data also suggests that further genome sequencing of the cyanobacterial phylum will widen the NRPS/PKS pathway diversity. Finally, genome mining leads to a better understanding of the functions of some of the secondary metabolites for the producing organisms and unveil new biosynthetic mechanisms that will become an inspiration for synthetic biology and biotechnical applications.

## Methods

### Dataset and strains

The 126 cyanobacterial genomes of the CyanoGEBA dataset [1] were retrieved from public databases (Additional file 1: Table S4). All genomes were reannotated using the MicroScope platform [56] with the exception of the genomes of *Nostoc azollae* 0708, *Crocosphaera watsonii* WH0003 and *Arthrospira platensis* Paraca, which were too fragmented. The PCC strains used in this study are available at Pasteur Culture collection of Cyanobacteria (http://cyanobacteria.web.pasteur.fr).

### Detection of genes coding for natural products

Natural product biosynthesis gene clusters were identified using a combination of the genome mining softwares met2db [57], antiSMASH [58], and NaPDoS [59]. Adenylation domain substrate specificity predictions for NRPS enzymes were made using NRPSpreditor2 [60]. Annotations were refined manually using CD-search [61], BLASTP [62] and InterProScan [63] to identify conserved domains. We estimated the number of gene clusters for each genome using the three methods. The NRPS modules encoded in typical modular NRPS gene clusters contained at least adjacent condensation (C) and adenylation (A) domains, and the NRPS-like clusters which lack the C domain were encompassed with the NRPS clusters as they were proved to actively produce secondary metabolite without a proper C domain [22]. The PKS type comprised at minimum a ketosynthase (KS) domain. The hybrid type comprised combinations of NRPS and PKS modules. The borders of each cluster were manually refined while checking for synteny among genomes using the MicroScope platform interface. In unfinished genomes, this method permitted also to find families of related gene clusters when the latter were fragmented on different contigs as one might expect.

### Cluster family reconstruction

All protein sequences of the clusters were compared against each other using the BLASTP (e-values ≤1e-20, identity ≥50%) and subsequently clustered using a transitive link criterion to build the cluster families (CFs) respecting the two following conditions: (i) two gene clusters belong to the same family if at least 80% of the gene content of the smallest cluster is shared with the larger gene cluster, (ii) two genes were considered to be related if their identity was greater or equal to 50% and if they aligned over at least 80% of their length. Some clusters split onto different contigs were reconnected into a single gene cluster during the cluster family reconstruction process.

### Hierarchical clustering

A Hierarchical Clustering analysis on the presence/absence pattern of all CFs found in the cyanobacterial genomes was done using the MeV software (v4.8; http://mev-tm4.sourceforge.net) and the following parameters: Pearson correlation, ordering optimization on the species, average linkage clustering.

### Species tree phylogeny

The species tree was generated by a concatenation of twenty-nine conserved proteins selected from the phylogenetic markers proposed for bacterial genome trees [64]. Homologs of each ribosomal protein were identified using BLASTP searches in the 126 cyanobacterial genomes as well as four outgroup genomes (*Chloroflexus auranticus* J-10, *Rhodobacter sphaeroides* 2.4.1, *Heliobacterium modesticaldum* Ice1, and *Chlorobium tepidum* TLS) and aligned using MAFFT v6.882b (default parameters) [65]. Ambiguous and saturated aligned regions were removed using the BMGE software 1.1 (parameter gap rate set to 0.5) [66]. The resulting twenty-nine alignments were then concatenated. A Maximum-Likelihood phylogenetic tree was generated with the alignment using PhyML 3.1.0.2 [67] using the LG amino acid substitution model with gamma-distributed rate variation (six categories), estimation of a proportion of invariable sites and exploring tree topologies using Nearest Neighbor Interchanges. 100 bootstrap replicates were performed.

### Domain phylogeny

472 KS and 939 C domain sequences predicted in the clusters were extracted from our dataset, alignments were generated and treated as described above, and used to generate for each a Maximum-Likelihood phylogenetic tree using the JTT amino acid substitution model with gamma-distributed rate variation (four categories), estimation of a proportion of invariable sites and exploring tree topologies using Nearest Neighbor Interchanges. 100 bootstrap replicates were performed for each dataset.

The sequence of 132 KS domains predicted to be involved in biosynthesis of polyunsaturated fatty acids and enediynes in our dataset was extended with 85 bacterial sequences from the secondary lipids dataset described previously [33]. The 217 sequences were aligned and filtered as described above. A maximum-likelihood phylogenetic tree with 100 bootstrap replicates was performed as for the Species tree.

### Sequence similarity search

Protein sequences of known secondary metabolite pathways were extracted from the NCBI web site according to the references for each cluster. These sequences were then compared with the proteins of the 126 genomes through BLASTP searches [62]. Protein sequences of all clusters were compared against the non-redundant database of the NCBI (April 2013) with the BLASTP in order to detect homologs (evalue ≤ 1e-20, identity ≥ 30%).

### Genomic context exploration

In order to explore the genomic context of each cluster, the gene composition of the cluster and the 10-kb flanking regions were analysed. First by processing genome annotations with multiple keywords, we identified common genetic elements involved in DNA mobility, *i.e.* phage, integrase or transposase. In addition to identify potential horizontally transferred genes in the genomes, we identified genes harbouring a +/- 1.5 time GC% standard deviation compared to the mean GC content and we computed the dinucleotide average absolute relative abundance difference (δ*-differences) between each cluster and the genome sequence as defined by Karlin (1998) [30]. Secondly, genome annotations were processed to identify genes linked to iron metabolism or iron transport related genes, *i.e*. *tonB* family genes, *fec/fhu* genes, siderophore transport systems, iron(III) dicitrate ABC transporter, *exbB/exbD* export system. If at least one of these genes was identified in the genomic context of each cluster, we hypothesized that the cluster could be involved in the synthesis of a potential iron siderophore.

### Anatoxin-a and microcystin detection

40 mg of freeze dried *Cylindrospermum* sp. PCC 7417 cells and 15 mg of freeze dried *Fischerella* sp. PCC 9339 cells were used for the detection of anatoxin-a and microcystin variants, respectively, using mass spectrometry as described previously [26, 68].

## Availability of supporting data

The data sets supporting the results of this article are available in the Dryad Digital Repository, http://doi.org/10.5061/dryad.p680f.

## Electronic supplementary material

Additional file 1: Table S1: 452 NRPS/PKS gene clusters, type, size, cluster family, genomic localization, putative siderophore gene clusters, dinucleotide average absolute relative abundance, percentage of genes deviated in GC% and mobility, **Table S2.** 20% of gene clusters involved in the production of known end-products in the 126 genomes, **Table S3.** The cluster families (CF) shared by several Cyanobacteria, and **Table S4.** Cyanobacterial strains of the CyanoGEBA dataset and the characteristics of the genomes studied. (PDF 3 MB)

Additional file 2: FigureS1: Maximum Likelihood phylogeny of all cyanobacteria included in this study, **Figure S2.** Detection of cyanotoxins in selected cyanobacteria containing toxin biosynthetic gene clusters, **Figure S3**. Abundance of the CFs in 89 cyanobacterial strains and comparsion of size of the shared and orphan CFs in finished and unfinished genomes, **Figure S4.** Maximum-likelihood phylogenetic tree of the 939 C domains detected in the 452 gene clusters, and **Figure S5.** Examples of secondary metabolite biosynthetic gene cluster families. (XLSX 76 KB)

Additional file 3: **Contains the hierarchical clustering on the presence/absence of the 286 NRPS/PKS gene cluster families in the 126 cyanobacterial genomes.** The left tree represents the Cluster Families detailed on the same line on the right. The tree at the top clusters the 126 genomes. A black square indicates the presence of a CF in a specific genome. Genomes possessing the same array of cluster families (CF-8/CF-58, CF-20, CF-1/CF-2) are grouped together. (JPEG 1 MB)

Additional file 4: **Contains the maximum-likelihood phylogenetic tree of the 939 C domains detected in the 452 gene clusters.** 100 bootstrap replicates were performed. The names of the leaves at the tip of each branch are indicated as follow: Strain number as in Table S1_genome ID in our database_protein ID_cluster type_domain type_domain begin_domain end_: length branch. (TXT 69 KB)

Additional file 5: **Contains the maximum-likelihood phylogenetic tree of the 472 KS domains detected in the 452 gene clusters.** 100 bootstrap replicates were performed. The names of the leaves at the tip of each branch are indicated as follow: Strain number as in Table S1_genome ID in our database_protein ID_cluster type_domain type_domain begin_domain end_: length branch. (TXT 31 KB)

## Abbreviations

A: Adenylation
Adda: 3-amino-9methoxy-2,6,8-trimethyl-10 phenyl-4,6-decadienoic acid
C: Condensation
CF: Cluster family
HGT: Horizontal gene transfer
KS: Ketoacyl synthase
NRPS: Non-ribosomal peptide synthetase
PKS: Polyketide synthase
PUFA: Polyunsaturated fatty acids.
