# Supplementary material for: Phylum-wide comparative genomics unravel the diversity of secondary metabolism in Cyanobacteria
Source: BMC Genomics. 2014 Nov 18;15(1):977. doi: 10.1186/1471-2164-15-977 (PMC4247773; doi:10.1186/1471-2164-15-977)
Supplement: Supplementary file 1 — Additional file 1: Table S1: 452 NRPS/PKS gene clusters, type, size, cluster family, genomic localization, putative siderophore gene clusters, dinucleotide average absolute relative abundance, percentage of genes deviated in GC% and mobility, Table S2. 20% of gene clusters involved in the production of known end-products in the 126 genomes, Table S3. The cluster families (CF) shared by several Cyanobacteria, and Table S4. Cyanobacterial strains of the CyanoGEBA dataset and the characteristics of the genomes studied. (PDF 3 MB) [file 12864_2014_6690_MOESM1_ESM.pdf]

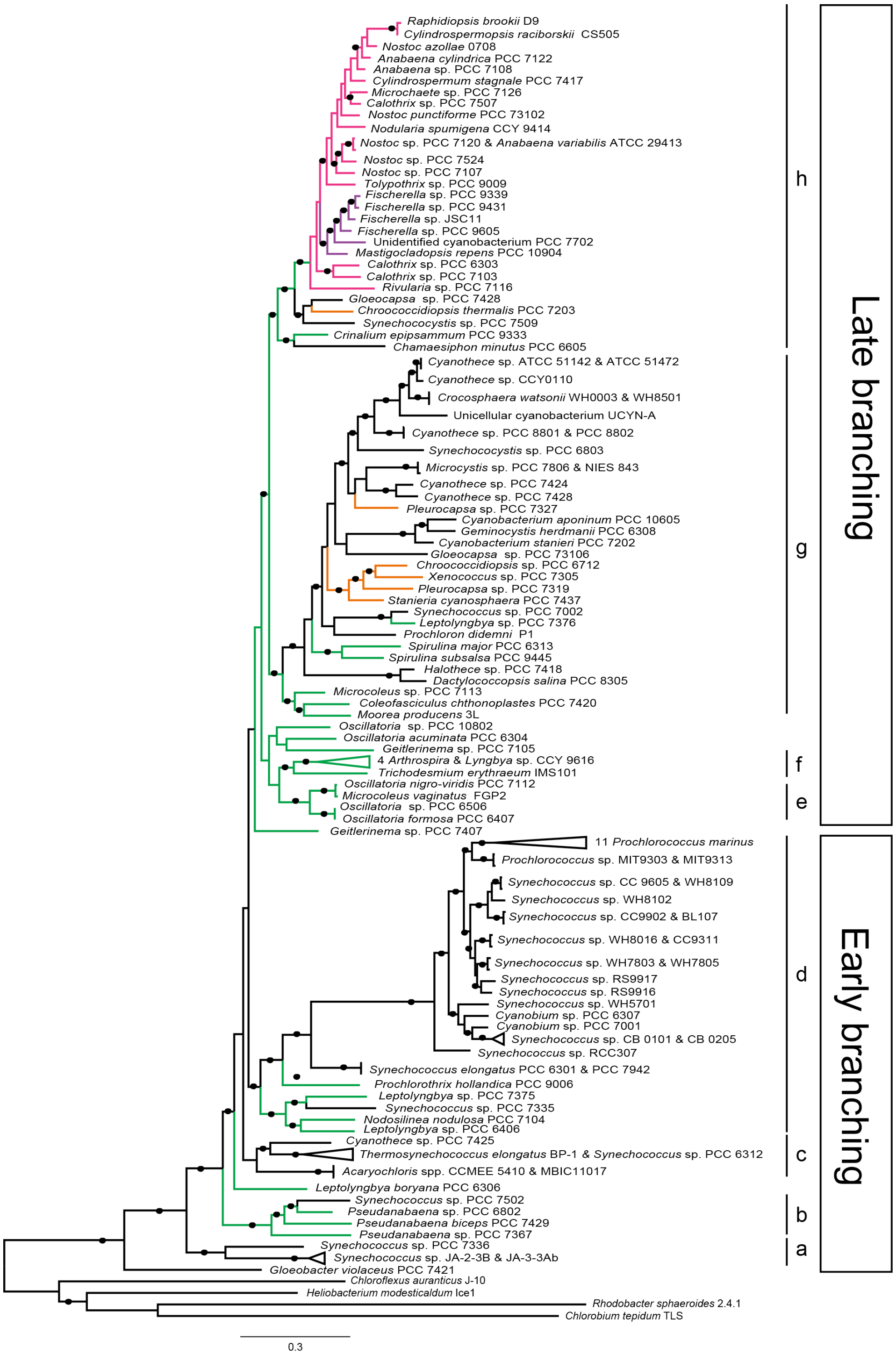

Figure S1. Maximum Likelyhood phylogeny of all cyanobacteria included in this study. The species tree was generated by a concatenation of twenty-nine conserved proteins (DnaG, Frr, NusA, Pgk, PyrG, RplA, RplB, RplC, RplD, RplE, RplF, RplK, RplL, RplM, RplN, RplP, RplS, RplT, RpmA, RpoB, RpsB, RpsC, RpsE, RpsI, RpsJ, RpsK, RpsM, RpsS and SmpB) selected from the phylogenetic markers proposed for bacterial genome trees [1]. Branches are color-coded based on morphology: black, unicellular; orange, unicellular with baeocyte; green, filamentous; pink; filamentous heterocystous; violet, filamentous heterocystous with ramification. Nodes supported with a bootstrap of ≥70% are indicated by a black dot. Seven phylogenetic subclades are distinguished, four in the late branching and tree in the early branching.

a1.

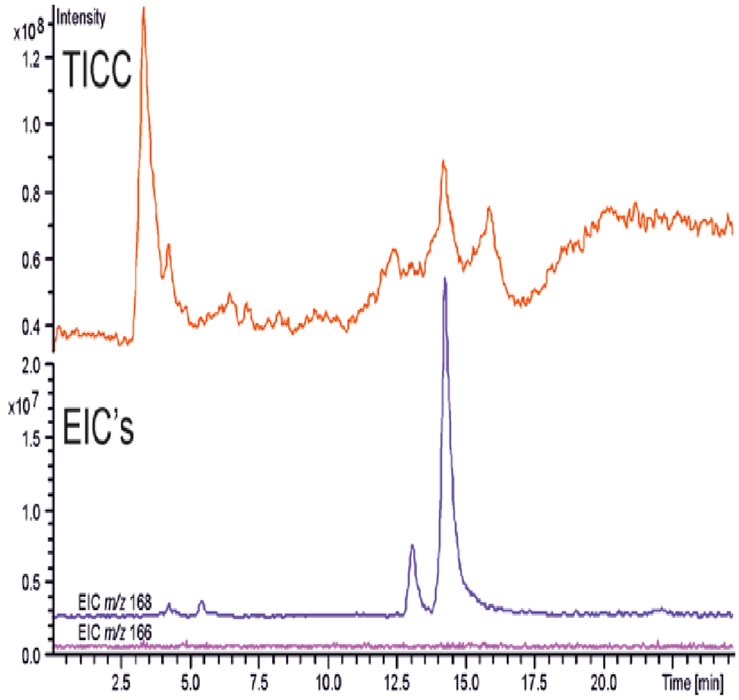
Intensity
x10^8
1.2
1.0
0.8
0.6
0.4
TICC
2.0
x10^7
1.5
1.0
0.5
0.0
EIC's
EIC m/z 168
EIC m/z 166
2.5
5.0
7.5
10.0
12.5
15.0
17.5
20.0
Time [min]

a2.

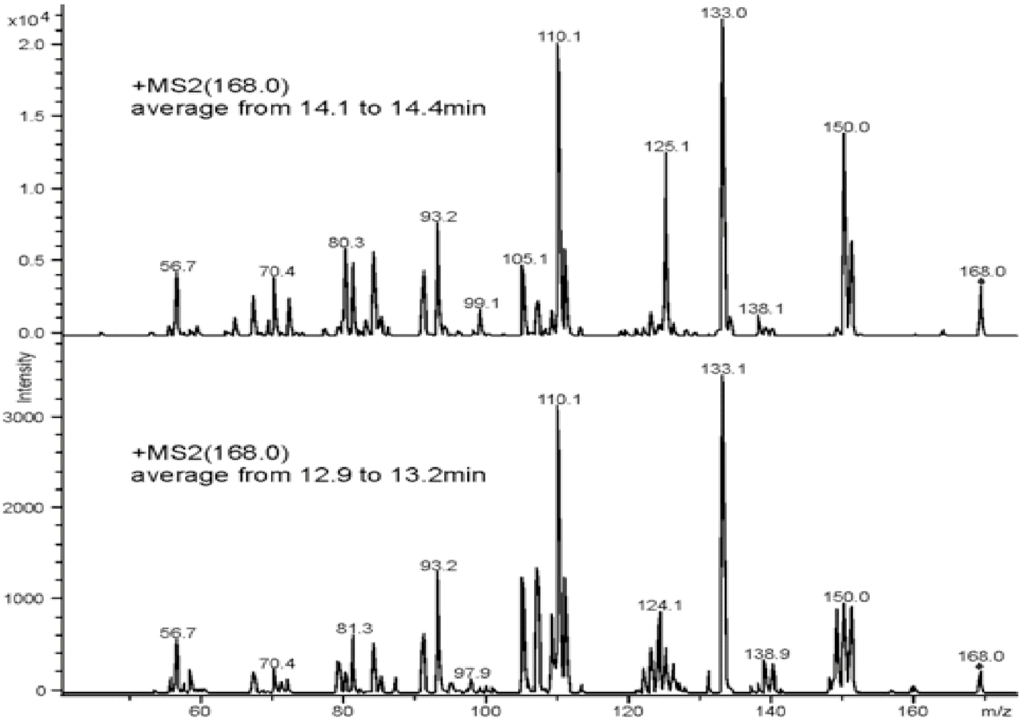
x10^4
2.0
1.5
1.0
0.5
0.0
+MS2(168.0)
average from 14.1 to 14.4min
56.7
70.4
80.3
93.2
99.1
105.1
110.1
125.1
133.0
138.1
150.0
168.0
Intensity
3000
2000
1000
0
+MS2(168.0)
average from 12.9 to 13.2min
56.7
70.4
81.3
93.2
97.9
110.1
124.1
133.1
138.9
150.0
168.0
60
80
100
120
140
160
m/z

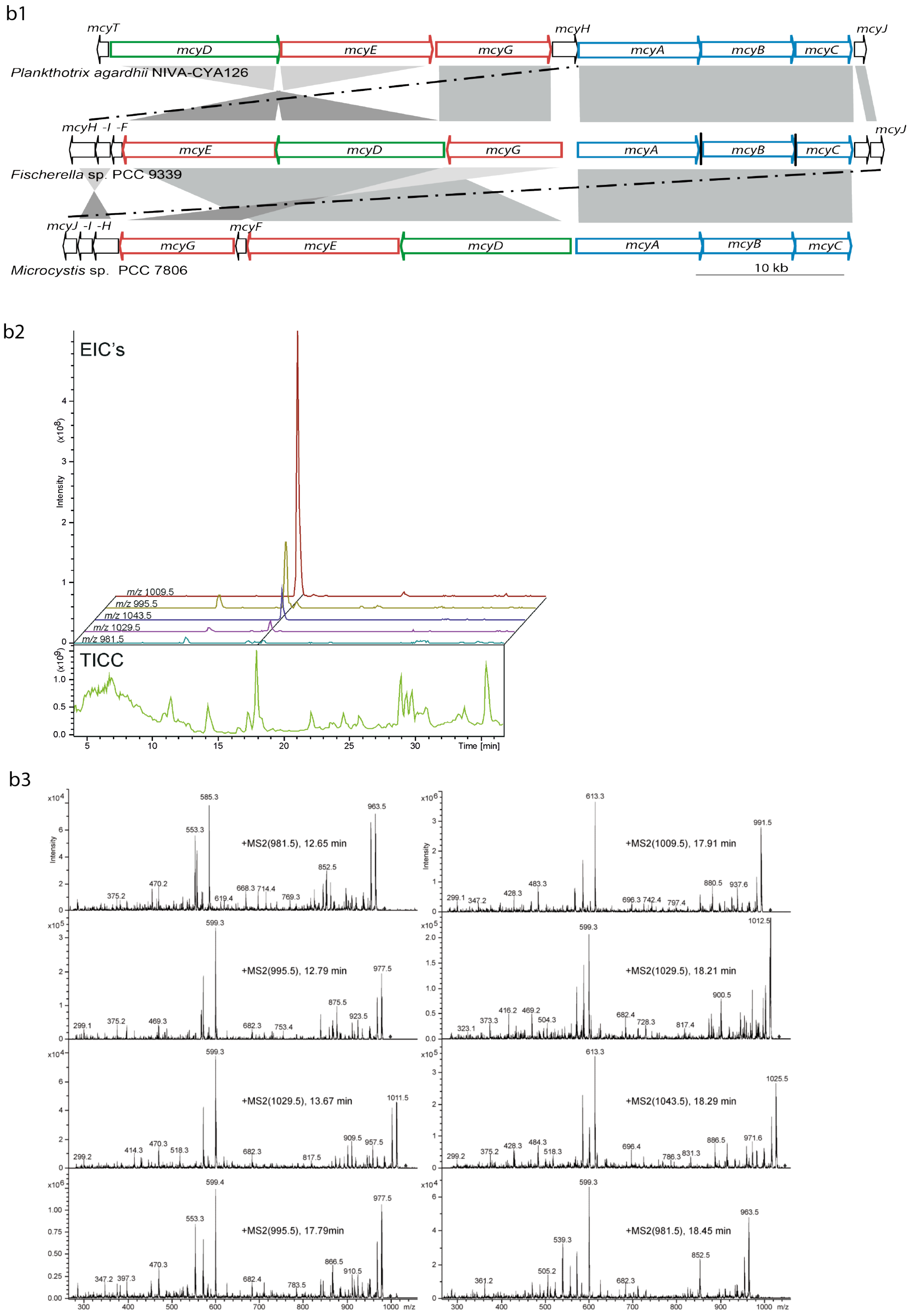

Figure S2. Detection of cyanotoxins in selected cyanobacteria containing toxin biosynthetic gene clusters. a. Dihydroanatoxin-a in *Cylindrospermum* sp. PCC 7417. a1,

Total ion current chromatogram (TICC) and extracted ion chromatograms (EIC's) corresponding to the protonated molecules of anatoxin-a (*m/z* 166.0) and dihydroanatoxin-a (*m/z* 168.0) from the extract of PCC 7417. Dihydroanatoxin-a was present in two forms (two peaks in EIC *m/z* 168). a2, Average product ion mass spectra of the protonated dihydroanatoxin-a molecules found in the extract of PCC 7417. Mass of the protonated dihydroanatoxin-a molecules and the retention time range (min) are presented with the spectrum. Both spectra show characteristic ion pattern for dihydroanatoxin-a previously determined[2]. b. Microcystin variants in *Fischerella* sp. PCC 9339. b1, Gene cluster for microcystin biosynthesis (CF-5 in this study) from the genome of *Fischerella* sp. PCC 9339 compared to homologous microcystin producing cyanobacteria [3, 4]. b2, Total ion current chromatogram (TICC) and extracted ion chromatograms (EIC's) corresponding to the protonated molecules (*m/z* 981.5, 1029.5, 1043.5, 995.5 and 1009.5) of the microcystins found in the methanol extract of the cyanobacterial strain PCC 9339. b3, Product ion mass spectra of the protonated microcystin molecules found in the methanol extracts of the cyanobacterial strain PCC 9339. Mass of the protonated microcystin molecules and the retention time (min) is presented with the spectrum. All spectra show the typical ion pattern and characteristic ions as *m/z* 361.2, 375.2, 539.3, 553.3 559.3 and 613.3 for microcystins when analysed with ion trap mass spectrometer.

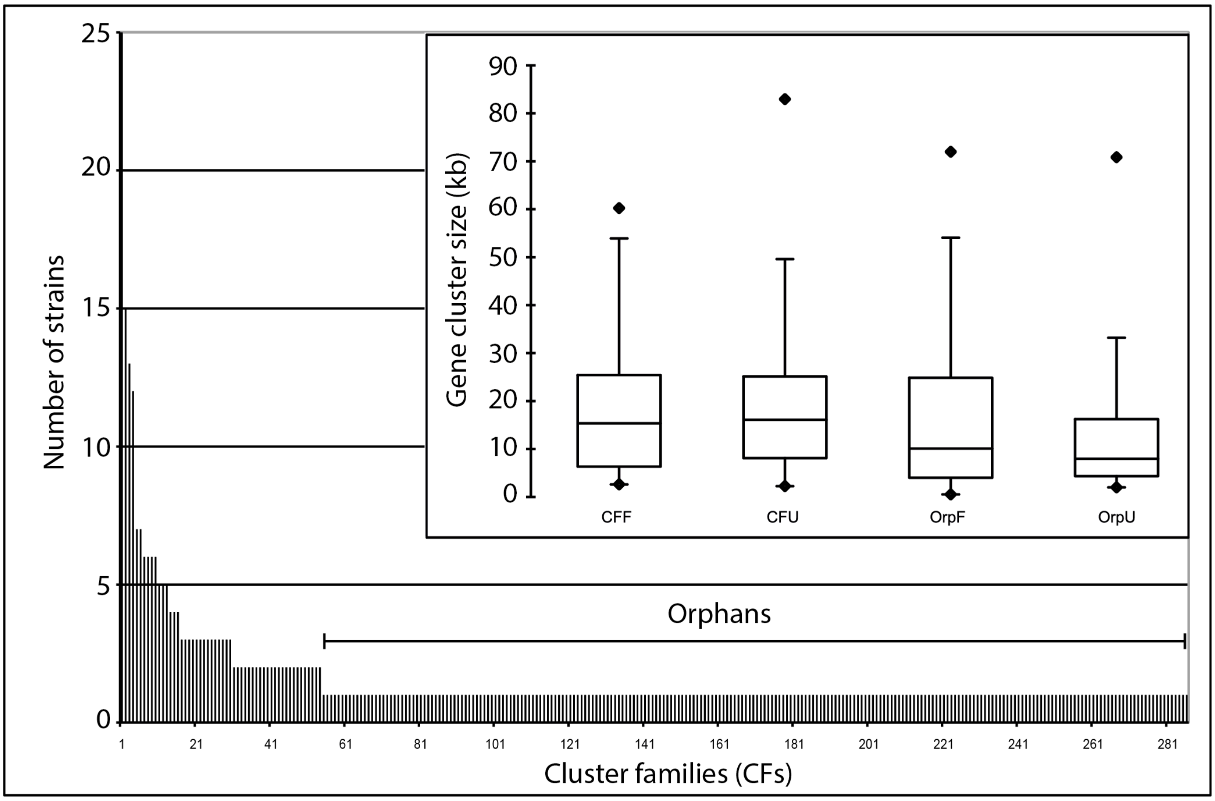

Figure S3. Abundance of the CFs in 94 cyanobacterial strains and comparsion of size of the shared and orphan CFs in finished and unfinished genomes. CFF, cluster family in complete genomes; CFU, cluster family in unfinished genomes, OrpF, orphan cluster in complete genomes, OrpU orphan cluster in unfinished genomes.

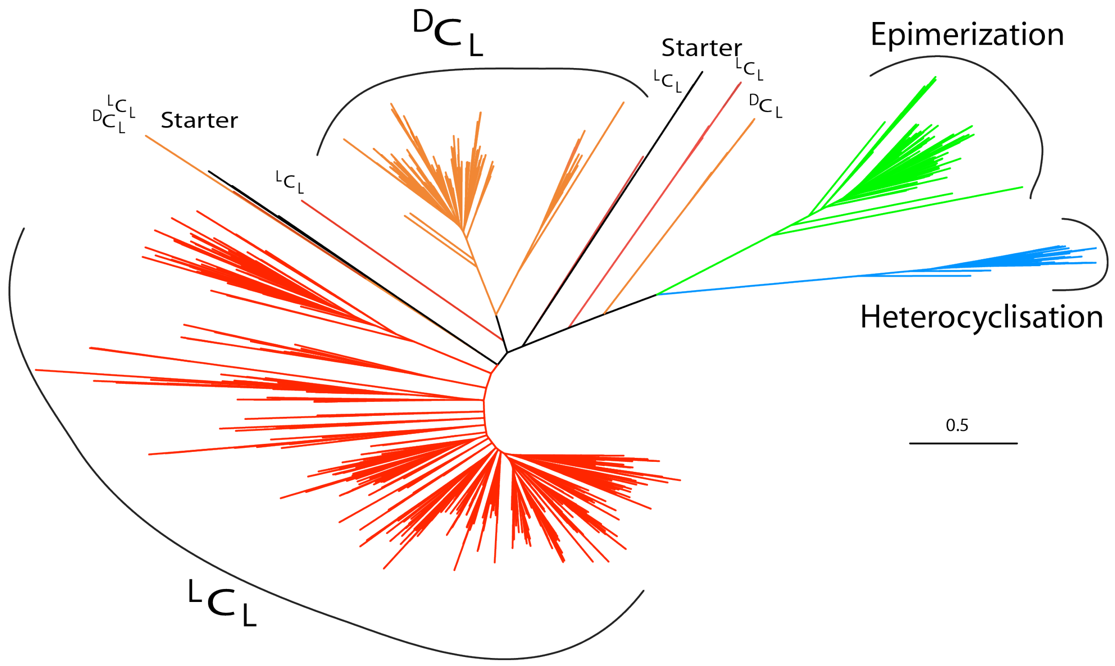

Figure S4. Maximum-likelihood phylogenetic tree of the 939 C domains detected in the 452 gene clusters. Branches are colored according to the C-domain type defined previously [5]: LCL-domains (red), DCL-domains (orange), Epimerization-domains (green), Heterocyclisation (blue) and Starter-domains (black). 100 bootstrap replicates were performed: all C-domain type groups were supported by high bootstrap values to the exception of LCL-type group. Although not supported, this latter group contains several well-supported (>80 %) subgroups. Note that the C-starter domains are separated into two groups and the high variability of subtypes of the C domains catalyzing a peptide bond between two L-amino acids and the ones linking L-amino acid to a growing peptide ending with a D-amino acid might represent more specialized subtypes capable of incorporating different types of substrates [5].

A. CF-20

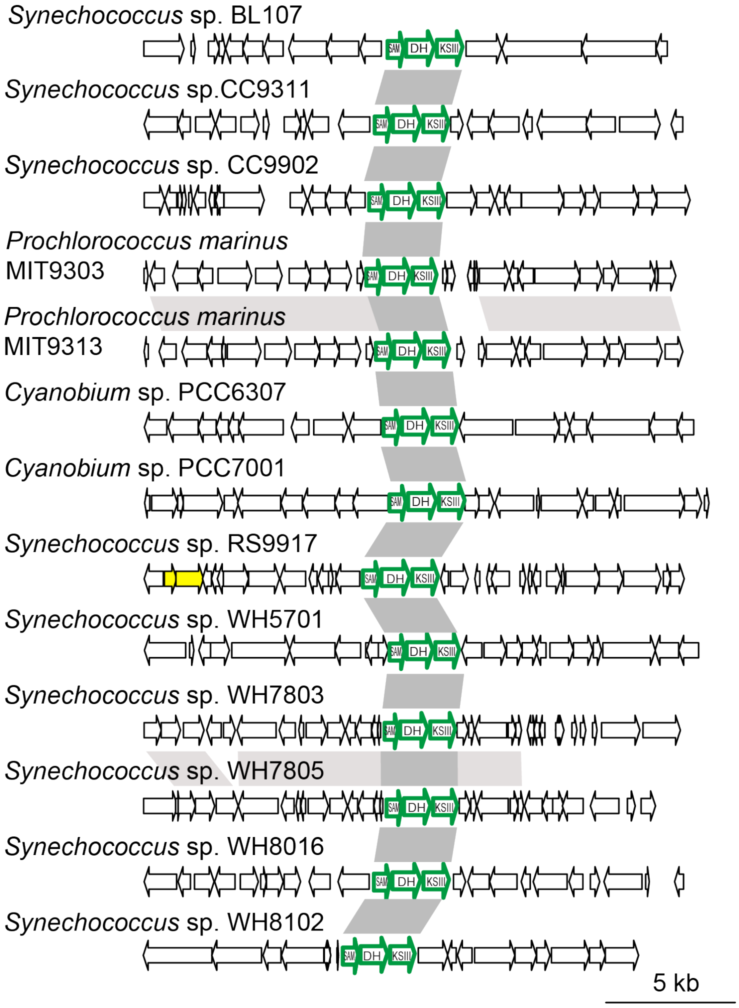

Synechococcus sp. BL107
Synechococcus sp.CC9311
Synechococcus sp. CC9902
Prochlorococcus marinus MIT9303
Prochlorococcus marinus MIT9313
Cyanobium sp. PCC6307
Cyanobium sp. PCC7001
Synechococcus sp. RS9917
Synechococcus sp. WH5701
Synechococcus sp. WH7803
Synechococcus sp. WH7805
Synechococcus sp. WH8016
Synechococcus sp. WH8102
SAM
DH
KSIII
5 kb

B. CF-26

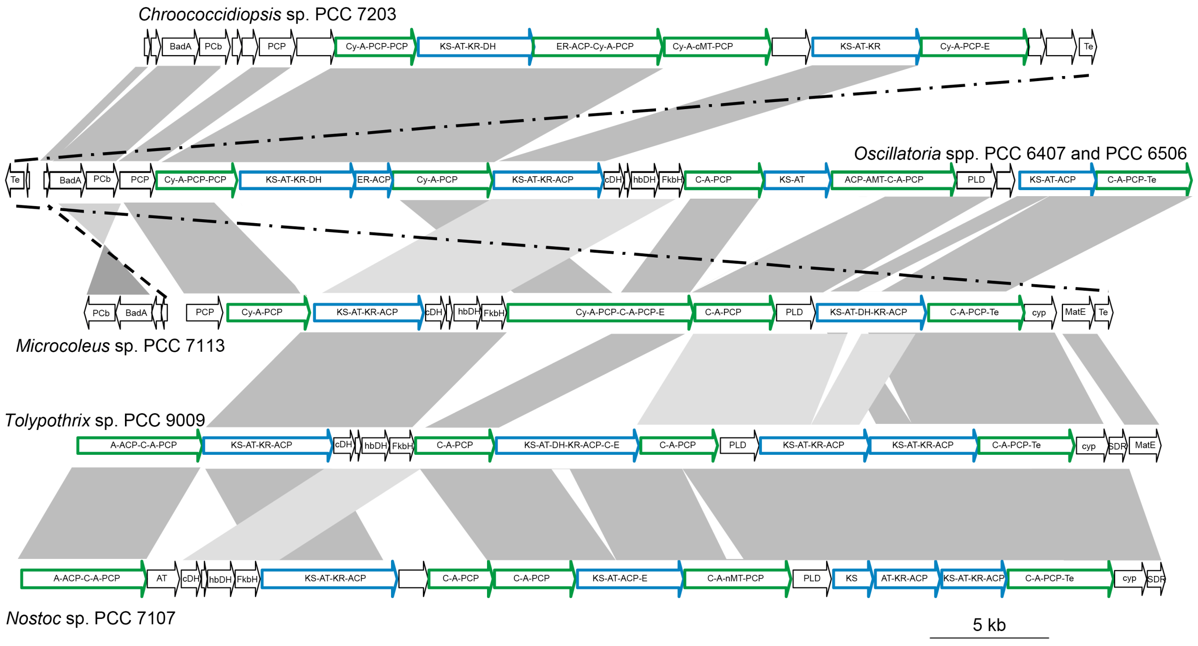

Chroococcidiopsis sp. PCC 7203
BadA
PCb
PCP
Cy-A-PCP-PCP
KS-AT-KR-DH
ER-ACP-Cy-A-PCP
Cy-A-cMT-PCP
KS-AT-KR
Cy-A-PCP-E
Te
Oscillatoria spp. PCC 6407 and PCC 6506
Te
BadA
PCb
PCP
Cy-A-PCP-PCP
KS-AT-KR-DH
ER-ACP
Cy-A-PCP
KS-AT-KR-ACP
cDH
hbDH
FkbH
C-A-PCP
KS-AT
ACP-AMT-C-A-PCP
PLD
KS-AT-ACP
C-A-PCP-Te
PCb
BadA
PCP
Cy-A-PCP
KS-AT-KR-ACP
cDH
hbDH
FkbH
Cy-A-PCP-C-A-PCP-E
C-A-PCP
PLD
KS-AT-DH-KR-ACP
C-A-PCP-Te
cyp
MatE
Te
Microcoleus sp. PCC 7113
Tolypothrix sp. PCC 9009
A-ACP-C-A-PCP
KS-AT-KR-ACP
cDH
hbDH
FkbH
C-A-PCP
KS-AT-DH-KR-ACP-C-E
C-A-PCP
PLD
KS-AT-KR-ACP
KS-AT-KR-ACP
C-A-PCP-Te
cyp
SDR
MatE
A-ACP-C-A-PCP
AT
cDH
hbDH
FkbH
KS-AT-KR-ACP
C-A-PCP
C-A-PCP
KS-AT-ACP-E
C-A-nMT-PCP
PLD
KS
AT-KR-ACP
KS-AT-KR-ACP
C-A-PCP-Te
cyp
SDR
Nostoc sp. PCC 7107
5 kb

C. CF-32

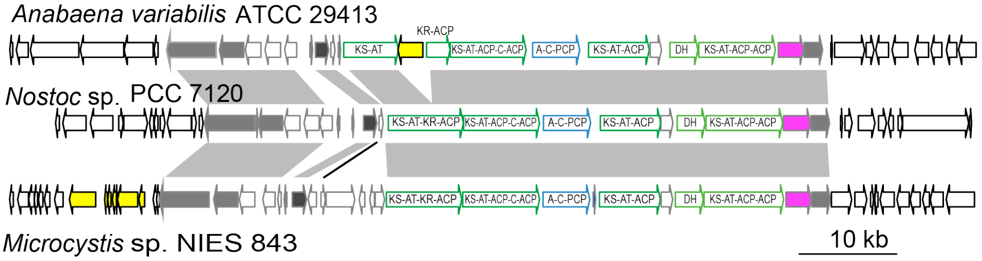

D. CF-45

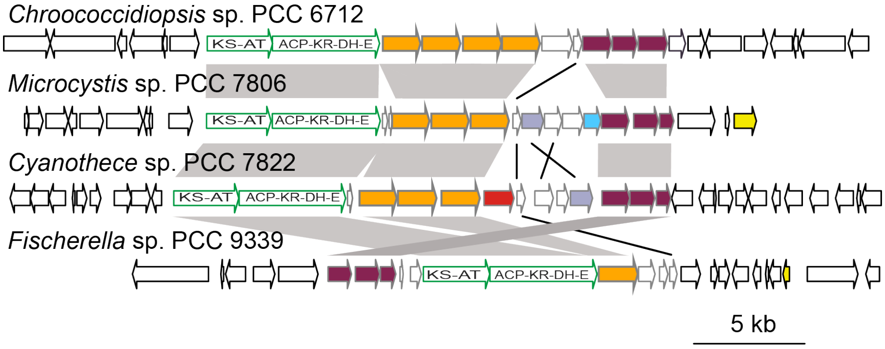

Figure S5. Examples of secondary metabolite biosynthetic gene cluster families. Genes with corresponding functions and domain organization are connected by grey areas, highlighting the genetic conservation and genetic rearrangements such as gene duplication/indel/inversion and domain deletion/substitution. The NRPS and PKS genes are outlined in blue and green, respectively, with the domains annotated as follow: SAM, S adenosyl methyltransferase; DH, dehydrogenase; KS, β-ketoacyl synthase; KSIII, β–ketoacyl synthase of type III; AT, acyl transferase; KR, ketoreductase; ACP, acyl carrier protein; C, condensation domain; A, adenylation; PCP, peptide carrier protein; AMT, aminomethyl transferase; cMT, carbon methyl transferase; nMT, nitrogen methyl transferase; Te, thioesterase; E, epimerase. BadA, benzoate-CoA ligase; PCb, putative chorismate binding domain protein; Cy, cyclisation; ER, enoyl reductase; PCP, peptidyl carrier protein; cDH, acyl-CoA dehydrogenase; hbDH, 3-hydroxyacyl-coA dehydrogenase; FkbH, 3-phosphoglyceryltransferase; PLD, pyridoxal dependent aspartate 1-decarboxylase; MatE, putative antimicrobial extrusion protein; cyp, putative cytochrome P450; SDR, short chain dehydrogenase/reductase. Genes encoding optional enzymes of these gene clusters or known functions are indicated as follow: grey, transporter; yellow, transposase; pink, oxygenase; red, hydrolase; blue, ATPase; orange, halogenase, purple oxidoreductase. The genes potentially involved in the gene cluster are outlined in grey, while the genes of the genomic context are outlined in black. A) CF-1 constituted of PKS of type III gene cluster conserved at > 48% in 13 picocyanobacterial genomes of the clade d. B) CF-26 constituted NRPS and PKS gene clusters and gene encoding optional enzymes. This gene cluster family is conserved at > 62% in six genomes across the cyanobacterial phylum C) CF-32 constituted of NRPS and PKS genes as well as genes encoding optional enzymes. This gene cluster family is conserved at > 72% in three cyanobacterial genomes and corresponds to the MIC1 biosynthetic cluster highly conserved in several *Microcystis* genomes [6]. D) CF-45 constituted of NRPS genes as well as genes encoding optional enzymes. This gene cluster family is conserved at > 54% in four cyanobacterial genomes and corresponds to the PKSI iterative biosynthetic cluster previously retrieved and highly conserved in *Microcystis* sp. PCC strains [4, 6].

References

1. Wu M, Eisen J: **A simple, fast, and accurate method of phylogenomic inference.** *Genome Biol* 2008, **9:**R151.
2. James K, Crowley J, Hamilton B, Lehane M, Skulberg O, Furey A: **Anatoxins and degradation products, determined using hybrid quadrupole time-of-flight and quadrupole ion-trap mass spectrometry: forensic investigations of cyanobacterial neurotoxin poisoning.** *Rapid Commun Mass Spectrom* 2005, **19:**1167-1175.
3. Christiansen G, Fastner J, Erhard M, Börner T, Dittmann E: **Microcystin biosynthesis in *Planktothrix*: genes, evolution, and manipulation. J Bacteriol.** *J Bacteriol* 2003, **185:**564-572.
4. Frangeul L, Quillardet P, Castets AM, Humbert JF, Matthijs HC, Cortez D, Tolonen A, Zhang CC, Gribaldo S, Kehr JC, et al: **Highly plastic genome of *Microcystis aeruginosa* PCC 7806, a ubiquitous toxic freshwater cyanobacterium.** *BMC Genomics* 2008, **9:**274.
5. Rausch C, Hoof I, Weber T, Wohlleben W, Huson D: **Phylogenetic analysis of condensation domains in NRPS in NRPS sheds light on their functional evolution.** *BMC Evol Biol* 2007, **7:**78.
6. Humbert JF, Barbe V, Latifi A, Gugger M, Calteau A, Coursin T, Lajus A, Castelli V, Oztas S, Samson G, et al: **A tribute to disorder in the genome of the bloom-forming freshwater cyanobacterium *Microcystis aeruginosa*.** *PLoS One* 2013, **8:**e70747.
