# Supplementary figures and images for: Phylum-wide comparative genomics unravel the diversity of secondary metabolism in Cyanobacteria
Source: BMC Genomics. 2014 Nov 18;15(1):977. doi: 10.1186/1471-2164-15-977 (PMC4247773; doi:10.1186/1471-2164-15-977)

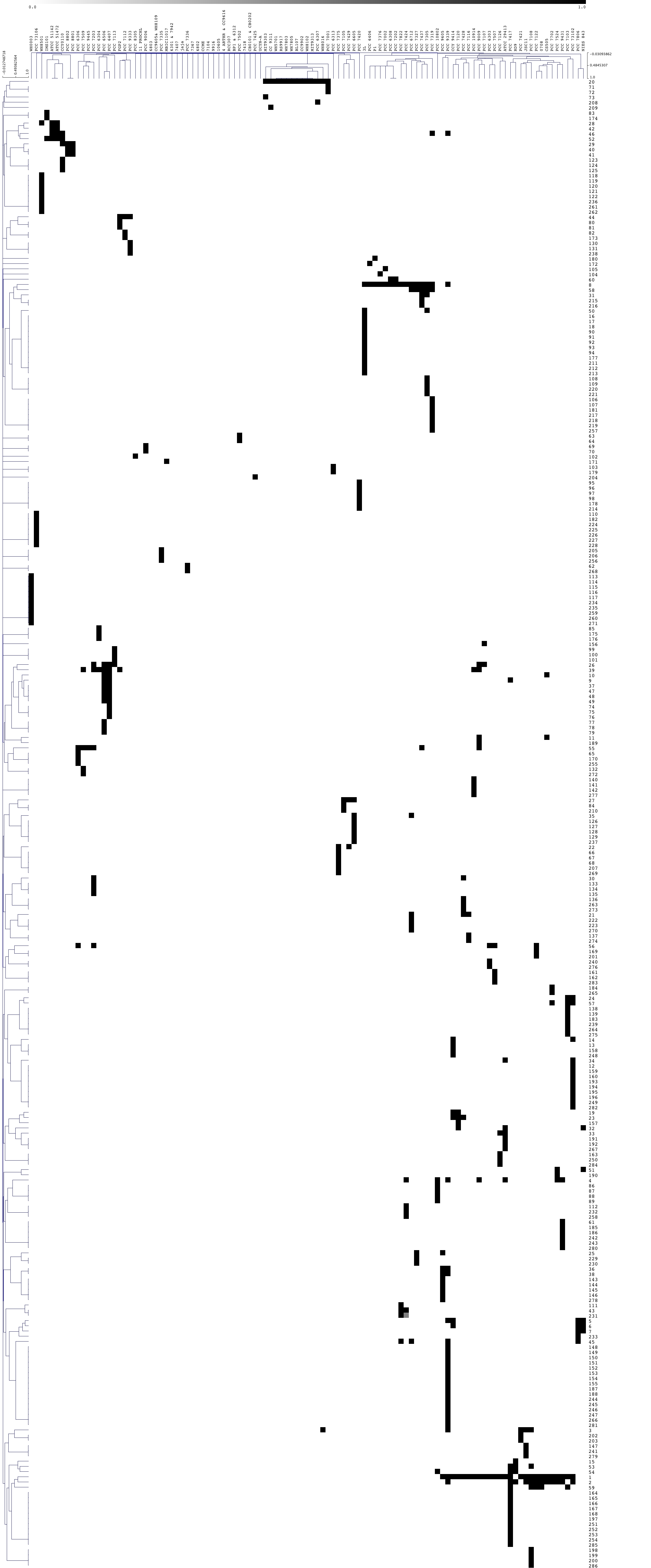

Supplement: Supplementary file 3 — Additional file 3: Contains the hierarchical clustering on the presence/absence of the 286 NRPS/PKS gene cluster families in the 126 cyanobacterial genomes. The left tree represents the Cluster Families detailed on the same line on the right. The tree at the top clusters the 126 genomes. A black square indicates the presence of a CF in a specific genome. Genomes possessing the same array of cluster families (CF-8/CF-58, CF-20, CF-1/CF-2) are grouped together. (JPEG 1 MB) [file 12864_2014_6690_MOESM3_ESM.jpeg]
